# Supplementary material for: The effective multiplicity of infection for HCMV depends on the activity of the cellular 20S proteasome
Source: J Virol. 2024 Dec 10;99(1):e01751-24. doi: 10.1128/jvi.01751-24 (PMC11784020; doi:10.1128/jvi.01751-24)
Supplement: Supplemental legends — Legends for Videos S1 to S4. [file jvi.01751-24-s0001.docx]

**SUPPLEMENTAL MATERIALS**

**Video S1 MOI 0.5 +BTZ.** MRC-5 fibroblasts infected with TB40-BAC4-derived HCMV expressing IE2-T2A-eGFP and pp28-mCherry at MOI 0.5 IU/cell with 15 nM BTZ.

**Video S2 MOI 0.5 +DMSO.** MRC-5 fibroblasts infected with TB40-BAC4-derived HCMV expressing IE2-T2A-eGFP and pp28-mCherry at MOI 0.5 IU/cell with DMSO.

**Video S3. MOI 3.0 +BTZ.** MRC-5 fibroblasts infected with TB40-BAC4-derived HCMV expressing IE2-T2A-eGFP and pp28-mCherry at MOI 3 IU/cell with 15 nM BTZ.

**Video S4. MOI 3.0 +DMSO.** MRC-5 fibroblasts infected with TB40-BAC4-derived HCMV expressing IE2-T2A-eGFP and pp28-mCherry at MOI 3.0 IU/cell with DMSO.
